# Radiomic features from multiparametric magnetic resonance imaging predict molecular subgroups of pediatric low-grade gliomas

**DOI:** 10.1186/s12885-023-11338-8

**Published:** 2023-09-11

**Authors:** Zhen Liu, Xuanke Hong, Linglong Wang, Zeyu Ma, Fangzhan Guan, Weiwei Wang, Yuning Qiu, Xueping Zhang, Wenchao Duan, Minkai Wang, Chen Sun, Yuanshen Zhao, Jingxian Duan, Qiuchang Sun, Lin Liu, Lei Ding, Yuchen Ji, Dongming Yan, Xianzhi Liu, Jingliang Cheng, Zhenyu Zhang, Zhi-Cheng Li, Jing Yan

**Affiliations:** 1 Department of Neurosurgery, The First Affiliated Hospital of Zhengzhou University, Jian she Dong Road 1, Zhengzhou, 450052 Henan province China; 2 Yanjing Medical College of Capital Medical University, Beijing, China; 3 Department of Pathology, The First Affiliated Hospital of Zhengzhou University, Zhengzhou, Henan China; 4 Department of MRI, The First Affiliated Hospital of Zhengzhou University, Jian she Dong Road 1, Zhengzhou, 450052 Henan province China; 5 Institute of Biomedical and Health Engineering, Shenzhen Institute of Advanced Technology, Chinese Academy of Sciences, Shenzhen, China; 6 University of Chinese Academy of Sciences, Beijing, China; 7 China-Japan Union Hospital of Jilin University, Changchun, Jilin China; 8 Shenzhen United Imaging Research Institute of Innovative Medical Equipment, Shenzhen, 518045 China

**Keywords:** Pediatric low-grade glioma, Magnetic resonance imaging, Radiomics, Machine learning, *BRAF* fusion

## Abstract

**Background:**

We aimed to develop machine learning models for prediction of molecular subgroups (low-risk group and intermediate/high-risk group) and molecular marker (*KIAA1549-BRAF* fusion) of pediatric low-grade gliomas (PLGGs) based on radiomic features extracted from multiparametric MRI.

**Methods:**

61 patients with PLGGs were included in this retrospective study, which were divided into a training set and an internal validation set at a ratio of 2:1 based on the molecular subgroups or the molecular marker. The patients were classified into low-risk and intermediate/high-risk groups, *BRAF* fusion positive and negative groups, respectively. We extracted 5929 radiomic features from multiparametric MRI. Thereafter, we removed redundant features, trained random forest models on the training set for predicting the molecular subgroups or the molecular marker, and validated their performance on the internal validation set. The performance of the prediction model was verified by 3-fold cross-validation.

**Results:**

We constructed the classification model differentiating low-risk PLGGs from intermediate/high-risk PLGGs using 4 relevant features, with an AUC of 0.833 and an accuracy of 76.2% in the internal validation set. In the prediction model for predicting *KIAA1549-BRAF* fusion using 4 relevant features, an AUC of 0.818 and an accuracy of 81.0% were achieved in the internal validation set.

**Conclusions:**

The current study demonstrates that MRI radiomics is able to predict molecular subgroups of PLGGs and *KIAA1549-BRAF* fusion with satisfying sensitivity.

**Trial registration:**

This study was retrospectively registered at clinicaltrials.gov (NCT04217018).

**Supplementary Information:**

The online version contains supplementary material available at 10.1186/s12885-023-11338-8.

## Background

Pediatric low-grade gliomas (PLGGs) are the most common pediatric brain tumors, accounting for more than 30% of central nervous system (CNS) tumors in children [1]. According to the 2016 World Health Organization (WHO) classification of CNS tumors, PLGGs comprise a histologically heterogenous group of Grade I and II tumors, including pilocytic astrocytoma (PA, Grade I), diffuse astrocytoma (AII, Grade II), oligodendroglioma (OII, Grade II), oligoastrocytoma (OAII, Grade II), pleomorphic xanthoastrocytoma (PXA, Grade II), dysembryoplastic neuroepithelial tumor (Grade I), neuronal-glial tumor (Grades I and II) and several others [2]. In clinical practice, PLGGs are generally regarded as a single group of tumors with relatively quiescent biological behavior and favorable prognosis [1–3]. Nevertheless, recurrence or progression still occurs in about 30% of PLGGs [1, 2, 4]. Postoperative adjuvant therapies for PLGGs include radiation therapy and systematic chemotherapy, which may cause long-term morbidity and toxicity [1].

Compared to adult low-grade gliomas, PLGGs have different features in molecular pathology [2]. Most PLGGs possess alterations in RAS/MAPK pathway, in which *BRAF* is a vital component [2, 5, 6]. In our previous study using a large set of 289 PLGGs to investigate biomarkers of molecular pathology and their clinical significance, the *KIAA1549-BRAF* fusion, *MYB* amplification, *CDKN2A* deletion, *BRAF*^*V600E*^, *H3F3A*, *TERT* promoter mutations, and *ATRX* loss were identified in PLGGs [2]. Emphatically, the combination of the previous molecular markers has successfully categorized PLGGs into four molecular risk groups (low-risk, intermediate-I, intermediate-II, and high-risk) with distinct survivals [2]. These findings highlight the importance of molecular stratification in evaluation and management of PLGGs.

Non-invasive prediction of molecular biomarkers or groups of gliomas is challenging [7]. Recent progress on artificial intelligence (AI) algorithms has considerably promoted automatically quantifying radiologic patterns, and several clinically relevant molecular biomarkers or groups have been identified by leveraging on AI algorithms in adult gliomas [8–11]. Recently, Wagner MW et al. developed and validated a radiomic signature that is predictive of the *BRAF* status of PLGGs [12]. However, there is a lack of study investigating the relationship between radiological features and risk groups of PLGGs defined by multiple molecular markers utilizing AI algorithms.

In the current study, radiomic features from multiparametric MRI, including T1-weighted, T1-weighted gadolinium contrast-enhanced, T2-weighted, fluid attenuated inversion recovery, and apparent diffusion coefficient images (T1, T1c, T2, FLAIR, and ADC), were extracted from 61 PLGG patients to construct models for prediction of molecular subgroups (low-risk group and intermediate/high-risk group) and molecular marker (*KIAA1549-BRAF* fusion) by leveraging machine learning algorithms. We aim to demonstrate that MRI patterns are significantly associated with key molecular biomarker and are able to predict molecular subgroups of PLGGs.

## Methods

### Patient enrollment

**Fig. 1:**
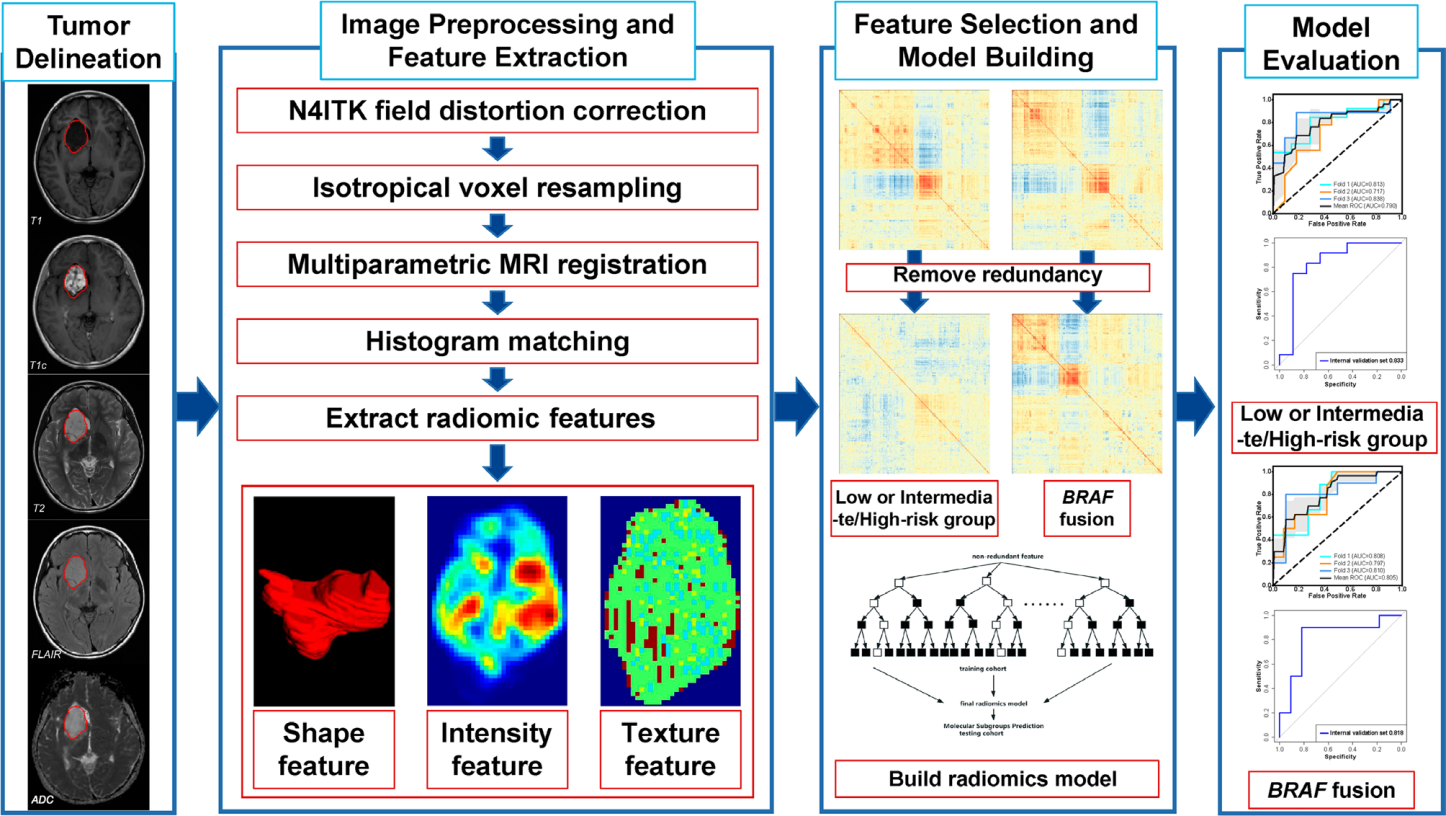
Overview of the radiomic pipeline. The pipeline consisted of tumor delineation, image preprocessing, radiomic feature extraction, feature selection, model building, and model evaluation

**Fig. 2:**
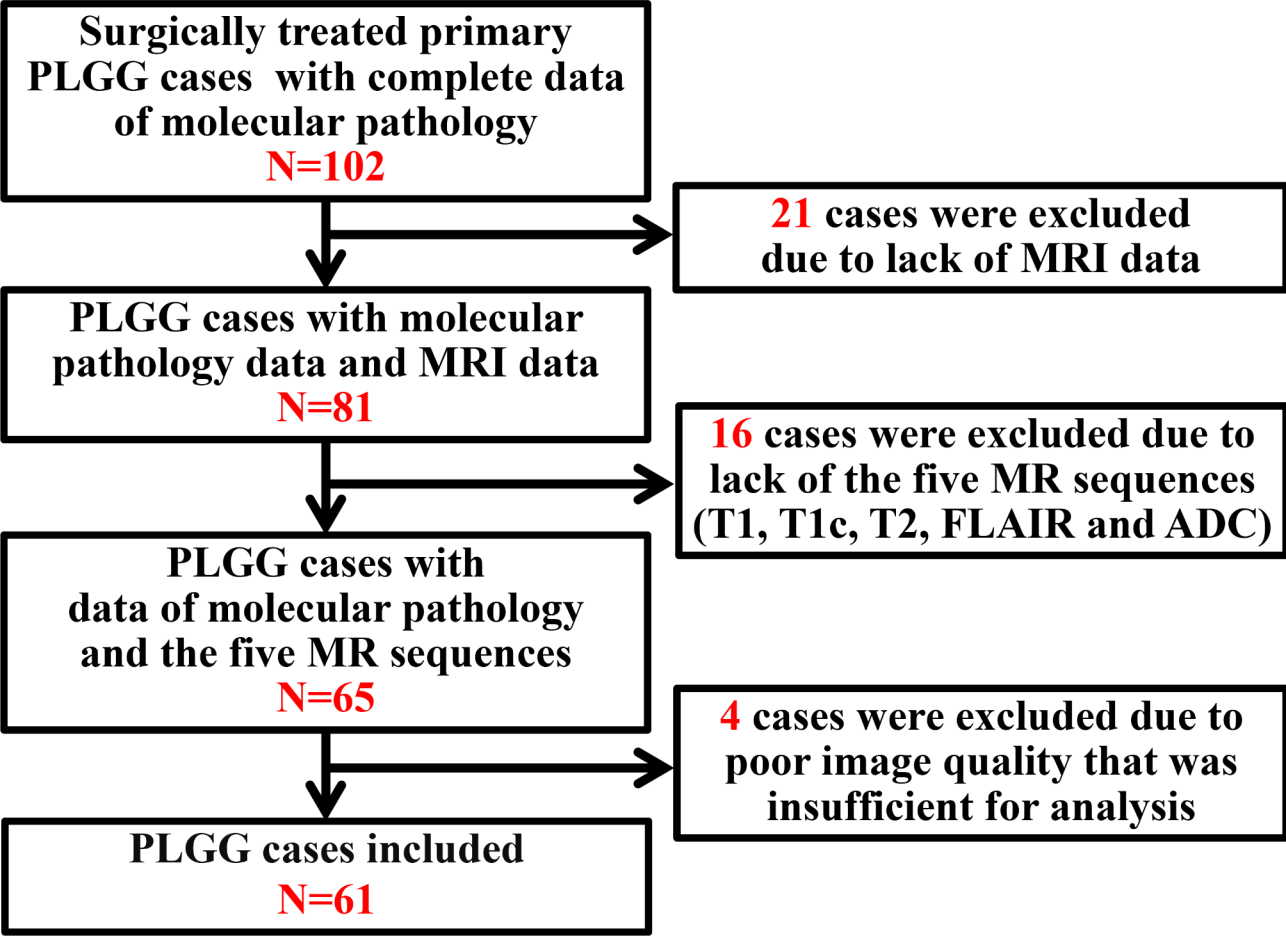
The patient selection process in this study

This study was a part of the registered clinical trial “MR Based Prediction of Molecular Pathology in Glioma Using Artificial Intelligence” (ClinicalTrials.gov ID: NCT04217018). The overview of the radiomic pipeline is illustrated in Fig. 1. This retrospective study was approved by the Human Scientific Ethics Committee of the First Affiliated Hospital of Zhengzhou University (No. 2019-KY-176), and the requirement for written informed consent was waived. 102 pediatric patients (Age<18) were diagnosed to have harbored primary PLGGs at the First Affiliated Hospital of Zhengzhou University (FAHZZU) between January 2011 and December 2016. The inclusion criteria for this study were: (1) newly diagnosed histologically confirmed PLGGs; (2) pretreatment MR imaging including T1, T1c, T2, FLAIR, and ADC; (3) MR images with sufficient image quality. After the patient enrollment process (Fig. 2), 61 patients were included in this study.

### MR imaging acquisition

All local MR images in FAHZZU were acquired on 1.5 T or 3.0 T clinical MR scanners, including Siemens, Philips, and GE Healthcare. The brain imaging protocol included the following sequences: (a) axial T1 before and after intravenous administration of a 0.1 mmol/kg dose of gadolinium-based contrast agent (T1c); (b) axial T2; (c) axial FLAIR; (d) diffusion-weighted imaging (DWI) and the corresponding ADC maps generated with the software incorporated into the MRI unit. Detailed information about the MR machines and imaging parameters are available in Supplementary Material and Supplementary Table 1.

### Detection of molecular pathology of PLGGs

Formalin-fixed paraffin embedded (FFPE) tissues were available in 102 cases. PCR and Sanger sequencing were performed to detect *TERT* promoter, *H3F3A*, *BRAF*, *FGFR1*, and *IDH1/2* mutations. *KIAA1549-BRAF* fusion, *MYB* amplification, and *CDKN2A* deletion were examined by Fluorescence in situ hybridization (FISH) analysis. Expression for ATRX and p53 proteins was evaluated by immunohistochemistry (IHC). Detailed protocols for the above molecular pathology were described by our previous research [2]. The molecular subgroups (low-risk and intermediate/high-risk) of PLGGs were assigned according to the integrative analysis of the above molecular markers [2]. Specifically, low-risk PLGGs were defined as patients with *BRAF* fusion or *MYB* amplification. Intermediate/high-risk PLGGs were defined as patients with *BRAF*^*V600E*^, and/or *CDKN2A* deletion, or *TERTp* mutation, or *H3F3A* mutation, or *ATRX* loss, or without alteration in any of the aboved biomarkers.

### Image preprocessing and lesion segmentation

Image preprocessing pipeline was carried out to normalize the intensity and geometry using an open-source tool ITK (https://itk.org/). First, N4ITK-based bias field distortion correction was performed. Then, all voxels were isotropically resampled into 1 × 1 × 1 mm^3^ with trilinear interpolation. The multiparametric MR images were co-registered to the corresponding axial resampled T1c with mutual information similarity metric, generating the registered images rT1, rT1c, rT2, rFLAIR, and rADC. Histogram matching was performed to normalize the intensity distribution. The volumes of interest (VOIs) were drawn manually slice-by-slice via the ITK-SNAP software (www.itksnap.org) by an experienced radiologist (J.Y. with 11 years’ experience) and consisted of the whole tumor regions (defined as the areas of abnormal signal on rFLAIR images). To assess the manual delineation-induced feature reproducibility, the VOI delineation process was repeated on 29 patients by another neurosurgeon (Z.Y.Z. with 11 years’ experience).

### High-throughput radiomic features extraction

All radiomic features were extracted using Pyradiomics extractor. To fully characterize the intra-tumor heterogeneity, three groups of features were extracted, including (1) shape features; (2) intensity features; and (3) texture features. Before feature extraction, fixed bin width of 25 was used for intensity discretization. The texture features were extracted using five different methods, including the gray-level co-occurrence matrix (GLCM), gray-level run length matrix (GLRLM), gray-level size zone matrix (GLSZM), gray-level dependence matrix (GLDM), and neighborhood gray-tone difference matrix (NGTDM). Two filters [wavelet transform, Laplacian of Gaussian (LoG) with four sigma levels (2.0, 3.0, 4.0, and 5.0)] were enabled in extracting intensity features and texture features from both the original images and the transformed images. Finally, 5929 quantitative features (14 volume and shape features, 1170 intensity features, and 4745 texture features) were extracted from multiparametric MRI (rT1, rT1c, rT2, rFLAIR, and rADC) for each patient. The extracted features were summarized in Supplementary Table 2. The YAML file for feature extraction can be found in Supplementary Material.

### Feature selection

Before feature selection, the stability of the extracted features was evaluated by interobserver reproducibility of the two image readers. We calculated the intraclass correlation coefficient (ICC) values between the same feature extracted from the two VOIs of 29 patients. The features with ICC value ≥ 0.90 were considered as highly reproducible features against the manual delineation and were retained for subsequent analysis while the others were discarded. Subsequently, all patients were randomly divided into three equal sets to realize 3-fold cross-validation. Two of these sets were fixed as the training set and the other was fixed as the internal validation set, which was repeated three times. Once the sample was split, we applied z-score normalization to the features in the training set. Then, the feature distribution in the training set was used to normalize the features in the internal validation set.

Feature selection was performed for the training set in the following two steps. First, the correlation coefficients between each pair of the remaining features were calculated in their own training set to minimize the feature redundancy. For a feature pair with correlation coefficients greater than 0.75, the feature with better univariate predictive power (smaller Mann–Whitney U test P value) was retained, while the other was removed. Second, based on the remaining robust and non-redundant feature subset, a random forest-based wrapper feature selection algorithm Boruta [13] was used to further select the optimal and relevant features in training set. Boruta searched for relevant features iteratively by comparing the importance of original features with the importance of artificially added random ones and progressively removing irrelevant features. After evaluating all possible feature combinations in the training set, the optimal features for the prediction model were selected.

### Machine learning classification

Based on the selected features, the radiomic models were built using a random forest algorithm for predicting the molecular subgroups (low-risk group and intermediate/high-risk group) and molecular marker (*KIAA1549-BRAF* fusion), respectively. An R package caret was used for random forest model building. In the random forest algorithm, the decision tree was constructed adopting the classification and regression tree (CART) method to classify the molecular subgroups and molecular marker, where the Gini index was used as importance measure [14]. After assessing a set of numbers ranging from 50 to 800, the number of trees in the random forest algorithm was set to 500. Here a 3-fold cross-validation was applied during model building. The data was partitioned into 3 folds, then 2 folds were used to train the model and the remaining fold was reserved for testing the model [15], and this process was repeated until the construction and evaluation of model was completed. Finally, the model of the best performance fold group of the 3-fold cross-validation scheme was chosen and validated.

### Statistical analysis

The enrolled patients were randomly divided into a training set and an internal validation set at a ratio of 2:1, where the distribution of clinical information was balanced. The differences in sex, age, molecular subgroups, and molecular marker between the training and internal validation sets were assessed by using the Wilcoxon test or Chi-square test. The classification performance was assessed in terms of the area under the receiver operating characteristic (ROC) curve (AUC), accuracy (ACC), sensitivity (SEN), specificity (SPE), and precision. We repeated the model using 3-fold cross-validation to achieve the best diagnostic performance and determine the final predictive model. The statistical analysis employed R studio software (R-4.0.5).

## Results

### Patient characteristics

Clinical and molecular characteristics of the 102 PLGGs surgically treated in FAHZZU were described in Supplementary Table 3. The models of optimal performance (risk group: fold 3; *BRAF* fusion: fold 3) in the cross-validation scheme were chosen. Patient characteristics of the training (n = 40) and internal validation sets (n = 21) were summarized in Table 1. There were no significant differences in the clinical and molecular characteristics between the training set and the internal validation set (*P* = 0.08-1.00).

**Table 1:** A summary of the clinical and molecular characteristics of patients (low-risk group and intermediate/high-risk group and *BRAF* fusion status) in PLGGs.

	Characteristic	Training set	Internal validation set	*P* Value
**Risk group**	Sex			0.33
	Male	20 (50.00%)	14 (66.67%)	
	Female	20 (50.00%)	7 (33.33%)	
	Age (year)			
	Mean ± SD	9.43 ± 4.93	9.81 ± 4.92	
	< 10	24 (60.00%)	11 (52.38%)	0.76
	≥ 10	16 (40.00%)	10 (47.62%)	
	Risk group			1.00
	Intermediate/High	16 (40.00%)	9 (42.86%)	
	Low	24 (60.00%)	12 (57.14%)	
***BRAF *** **fusion**	Sex			0.08
	Male	26 (65.00%)	8 (38.10%)	
	Female	14 (35.00%)	13 (61.90%)	
	Age (year)			
	Mean ± SD	9.38 ± 5.11	9.90 ± 4.54	1.00
	< 10	23 (57.50%)	12 (57.14%)	
	≥ 10	17 (42.50%)	9 (42.86%)	
	*KIAA1549-BRAF* fusion			0.91
	Yes	23 (57.50%)	11 (52.38%)	
	No	17 (42.50%)	10 (47.62%)	

### Feature selection

**Fig. 3:**
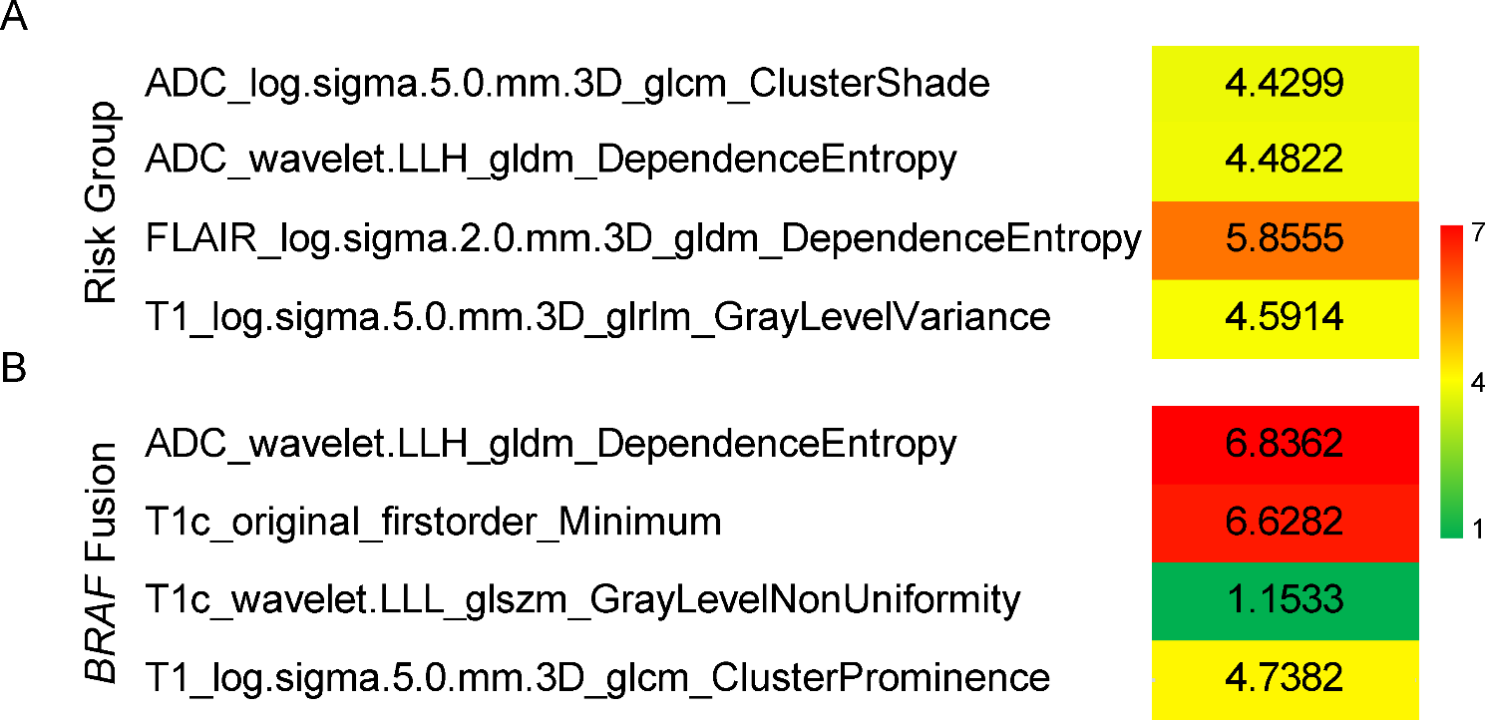
The Gini index of the selected features

After the ICC repeatability test and Boruta algorithm, four all-relevant texture features were selected for the classification of molecular subgroups. Meanwhile, four all-relevant features were selected for the prediction of *BRAF* gene fusion, including three texture features and one intensity feature. The feature selection results were summarized in Table 2. To evaluate the univariate contribution of these features to the prediction of molecular subgroups and *BRAF* fusion, we calculated the Gini index as the importance value (Fig. 3). A larger value indicates greater importance in predicting a specific subgroup. In addition, the meanings of these selected features are described in Supplementary Table 4.

**Table 2:** A summary of the selected all-relevant features

	Selected features	Type	Sequence	Transform
**Risk group**	ClusterShade	texture	ADC	log.sigma.5.0.mm.3D
	DependenceEntropy	texture	ADC	wavelet.LLH
	DependenceEntropy	texture	FLAIR	log.sigma.2.0.mm.3D
	GrayLevelVariance	texture	T1	log.sigma.5.0.mm.3D
***BRAF *** **fusion**	DependenceEntropy	texture	ADC	wavelet.LLH
	Minimum	intensity	T1c	original
	GrayLevelNonUniformity	texture	T1c	wavelet.LLL
	ClusterProminence	texture	T1	log.sigma.5.0.mm.3D

ADC: apparent diffusion coefficient; FLAIR: fluid attenuated inversion recovery; T1c: T1-weighted gadolinium contrast-enhanced; T1: T1-weighted

### Prediction of molecular subgroups and *BRAF* fusion

**Fig. 4:**
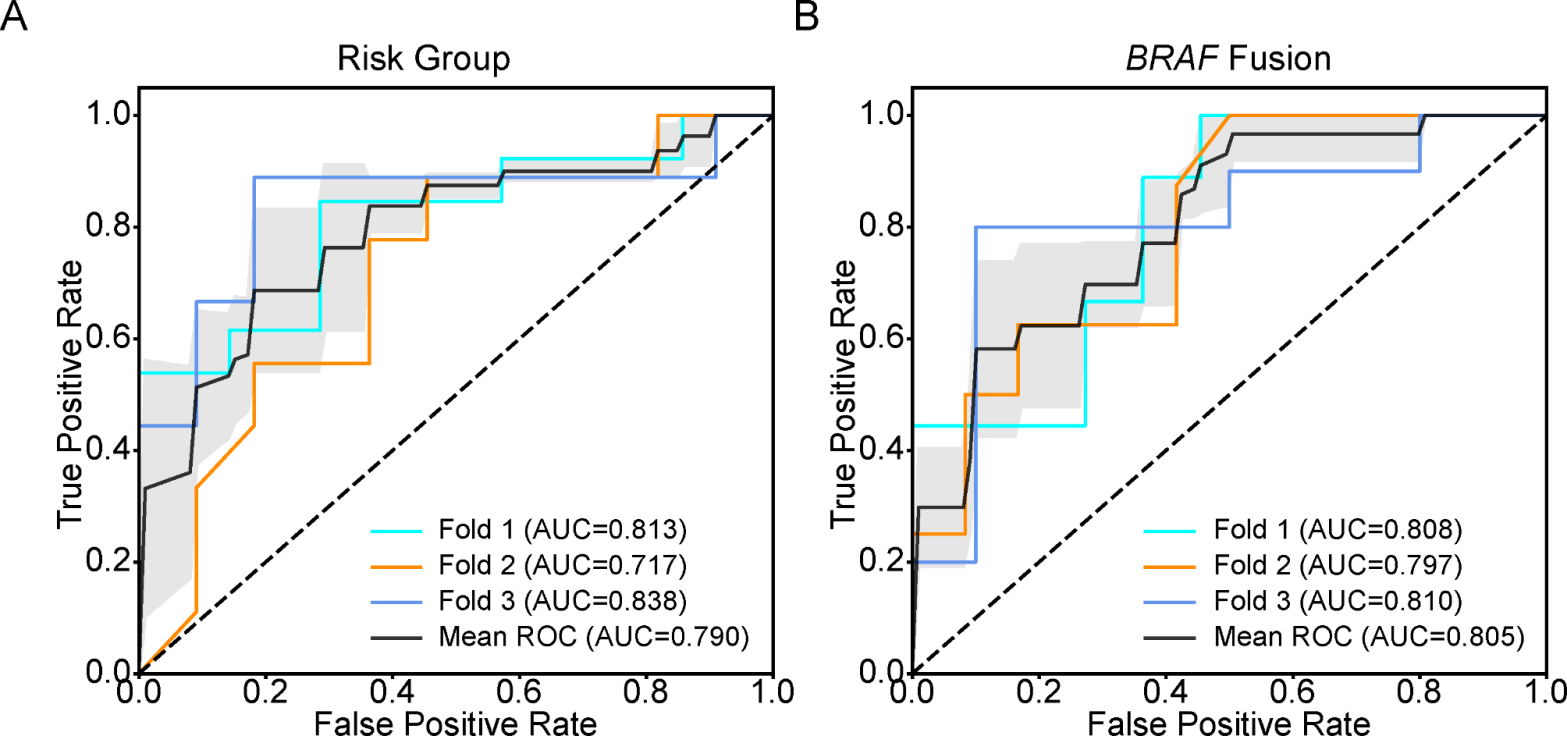
The ROC curves of the radiomic models with a 3-fold cross-validation scheme for classification of low-risk group and intermediate/high-risk group (**A**), and prediction of *BRAF* fusion (**B**)

**Fig. 5:**
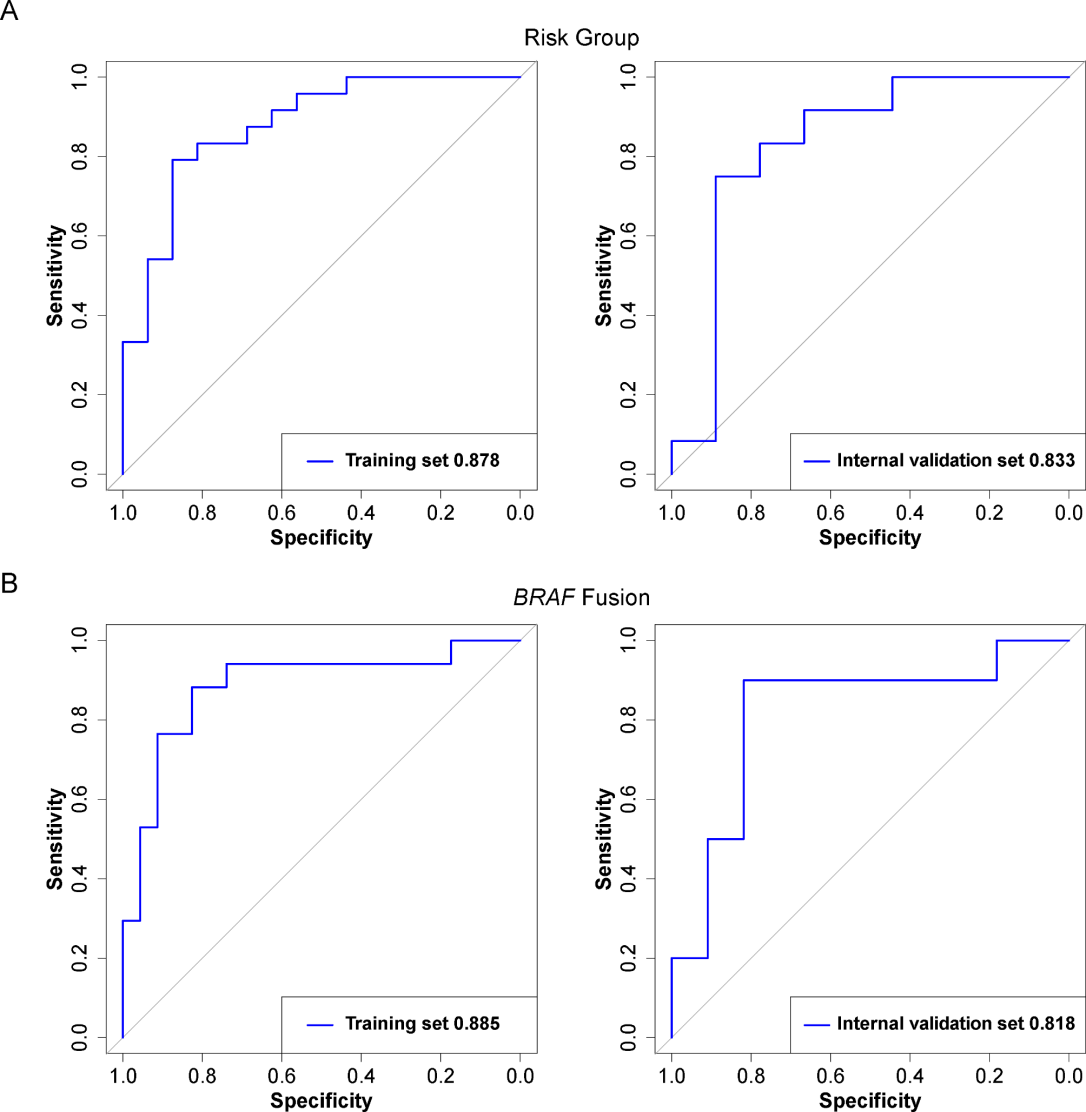
The ROC curves in both training and internal validation sets using the optimal hyperparameters obtained by 3-fold cross-validation

The ROC curves with a 3-fold cross-validation scheme to predict molecular subgroups and *BRAF* fusion are shown in Fig. 4. By means of the optimal hyperparameters obtained from fold 3, the ROC curves of the two prediction models in both training and internal validation sets are calculated and displayed in Fig. 5. The performance of the prediction models is summarized in Table 3.

**Table 3:** A summary of the subgroup-specific classification performance of the radiomic models

Molecular subgroups	Data Sets	AUC (95% CI)	ACC (%) (95% CI)	SEN (%) (95% CI)	SPE (%) (95% CI)	Precision (95% CI)
**Low-risk vs. intermediate/** **high-risk**	Training	0.878 (0.766, 0.989)	0.825 (0.700, 0.925)	0.792 (0.625, 0.917)	0.875 (0.688, 1.000)	0.909 (0.783, 1.000)
	Internal validation	0.833 (0.624, 1.000)	0.762 (0.550, 0.900)	0.714 (0.571, 0.905)	0.889 (0.667, 1.000)	0.900 (0.700, 1.000)
***BRAF*** **fusion positive** **vs. negative**	Training	0.885 (0.770, 1.000)	0.850 (0.725, 0.950)	0.882 (0.706, 1.000)	0.826 (0.652, 0.957)	0.800 (0.650, 0.941)
	Internal validation	0.818 (0.613, 1.000)	0.810 (0.666, 0.952)	0.900 (0.700, 1.000)	0.727 (0.455, 1.000)	0.750 (0.588, 1.000)

AUC: area under the curve; ACC: accuracy; SEN: sensitivity; SPE: specificity; CI: confidence interval

For the classification model of differentiating low-risk PLGGs from intermediate/high-risk PLGGs, the internal validation set yields an AUC of 0.833 (95% CI: 0.624-1.000). The ACC, SEN, SPE, and precision were 0.762 (95% CI: 0.550–0.900), 0.714 (95% CI: 0.571–0.905), 0.889 (95% CI: 0.667-1.000), and 0.900 (95% CI: 0.700-1.000), respectively.

For the prediction model of *KIAA1549-BRAF* fusion, the internal validation set yields an AUC of 0.818 (95% CI: 0.613-1.000). The ACC, SEN, SPE, and precision were 0.810 (95% CI: 0.666–0.952), 0.900 (95% CI: 0.700-1.000), 0.727 (95% CI: 0.455-1.000), and 0.750 (95% CI: 0.588-1.000), respectively.

## Discussion

As the most frequent brain tumors in children, PLGGs comprise a group of gliomas with heterogeneous histological types and different tumor locations [16, 17]. In the past decades, novel biological insights into the genetic background of PLGGs have been acquired by extensive investigations [5, 6, 18–21]. Unlike adult low-grade gliomas that are characterized by robust molecular alterations such as *IDH* mutations, 1p/19q codeletion, and *TERT* promoter mutations [22–24], PLGGs harbor their own molecular alterations distinct from adult counterparts [3]. It was revealed that nearly all PLGGs converge on the alterations of the MAPK pathway, with these alterations 100% existing in pediatric pilocytic astrocytoma [5, 6]. The most common molecular alteration in the MAPK axis is *KIAA1549-BRAF* fusion, which is caused by tandem duplication and rearrangement between *BRAF* and *KIAA1549* at chromosome 7q34 [2]. The *BRAF* gene is also the most common point mutation target in PLGGs, the majority being V600E hotspot mutation [19, 20]. Several studies have demonstrated that *KIAA1549-BRAF* fusion predicts better survivals in patients with PLGGs [2, 25], while *BRAF*^*V600E*^ point mutation is associated with inferior prognosis [26]. Aside from predictive values, *BRAF* gene alterations are also targets for novel drugs used in preliminary clinical trials for PLGGs. *BRAF* inhibitor such as dabrafenib has shown a positive response rate in a multicenter phase I study including patients with PLGGs [27]. Zhang J et al. reported 25% of diffuse cerebral gliomas in children carried abnormalities in *MYB* and *MYBL1* using whole genome sequencing [6]. Our previous study has identified *MYB* amplification in 10.6% PLGGs and revealed this genetic alteration was associated with significantly longer survivals of PLGGs. Therefore, the presence of either *KIAA1549-BRAF* fusion or *MYB* amplification categorizes a low-risk subset of PLGGs with a favorable prognosis [2].

Due to the limited number of intermediate (n = 23) and high-risk (n = 2) groups in the current set, we combined these two risk groups into one risk group (intermediate/high-risk group). Compared to the intermediate/high-risk group, the low-risk group of PLGGs confers an excellent survival with no mortality and rare tumor recurrence until the follow-up time point [2]. Therefore, identifying this group of PLGGs is of considerable significance since it has the potential to aid clinical decision-making such as the selection of molecular targeted therapies or curtailment of postoperative adjuvant therapies. However, the assignment of PLGGs risk groups requires the detection of multiple molecular biomarkers (*BRAF* fusion, *MYB* amplification, *BRAF*^*V600E*^ mutation, *CDKN2A* deletion, *TERTp* mutation, *H3F3A* mutation, and *ATRX* loss), which are not all available in a vast number of medical centers with constrained resources. In addition, postoperative rather than the preoperative assignment of risk groups of PLGGs will inevitably lose the chance to guide personalized surgical resection strategies for these tumors. For instance, in low-risk group of PLGGs, it may be wise to perform palliative resection to preserve neurological function, rather than pursuing total tumor resection, since this group of PLGGs presents quiescent biological behavior.

Radiomics is an emerging research realm that investigates the relationship between radiographic features and tumor genotype, which serves as a promising approach to discriminate surrogate biomarkers with an accurate reflection of tumor genomics [7]. Radiographic data from MRI of CNS tumors are also extensively investigated by radiomic strategies by leveraging AI algorithms, and adult gliomas are the most frequently studied CNS tumors [7]. Specifically, machine learning or deep learning algorithms trained on preoperative MRI were demonstrated to predict molecular biomarkers such as *IDH* mutations, 1p/19q codeletion, and *TERT* promoter mutations, or molecular subgroups based on *IDH* mutations and 1p/19q codeletion in adult gliomas with remarkable sensitivity and specificity [8–11]. A previous study has revealed radiomics-based prediction of *BRAF* status in PLGGs appears feasible [12]. However, whether radiomic features could accurately reflect the risk group of PLGGs remains unexplored. Our results demonstrated preoperative MRI patterns were able to predict either molecular biomarker (*BRAF* fusion) or risk group based on multiple molecular biomarkers, and yielded a satisfying performance, with AUC of 0.818 and 0.833, respectively.

It is worth noting that, identifying accurate and reproducible radiomic features of tumors is an essential step before translating into clinical application. As described in the previous literature, we employed a two-reader manual delineation approach by calculating the ICC between the same feature extracted from two VOIs to assess the feature reproducibility [28–30]. With the advancement of computing power, the use of semi-automatic or automatic approach has also provided sufficiently reliable tumor segmentation and feature stability [12, 31, 32].

To our knowledge, no prior study has investigated radiomic features of PLGGs using DWI. Our results indicate that the GLCM or GLDM texture features of ADC maps contribute to assess either molecular alteration or risk stratification in PLGGs. Likewise, it was reported that texture features and ADC parameters were important imaging markers to discriminate molecular subtypes in adult diffuse gliomas [9, 33–35]. For instance, Kihira S et al. found that addition of GLCM texture features from diffusion images to conventional MRI features could improve the diagnostic performance in determination of *MGMT* methylation status in gliomas [35]. Meanwhile, in our previous study, the results showed that the GLCM texture features from ADC maps played an important role in predicting *IDH* mutation and *TERT* promoter mutation of gliomas, while GLDM texture features from ADC maps were important for 1p/19q codeletion [9]. These phenomena may partly be explained by, that ADC values of brain tumors are inversely related to cellularity [36], and that texture features quantify local image patterns reflecting subtle intratumoral heterogeneity [33]. In addition, features from the conventional MR sequences were also revealed to play a role in the prediction model for *BRAF* status or risk stratification of PLGGs. This may explain why in the internal validation set, we archived a higher AUC (mean AUC = 0.805) than the AUC (0.75) reported by the previous study using only FLAIR sequence for model development [12]. It is reasonable to infer that a radiomic signature with features from multiparametric MRI is more effective and reliable than a single sequence.

Several limitations need to be pointed out in the current study. The first limitation is the relatively small sample size of the set, which hampers us to divide the intermediate-risk group from high-risk group for developing prediction model. Multi-institutional studies with larger sample size are necessary to further validate our findings. Second, advanced MR sequences such as diffusion tensor imaging (DTI), perfusion-weighted imaging (PWI), and diffusion kurtosis imaging (DKI) are welcome to further excavate the potential of MRI for predicting genotypes of PLGGs. Third, extensive integrative analysis on high through-put sequencing with paired MRI data, as well as in vivo imaging studies are required to clarify the elusive mechanisms on the relationship between radiomic patterns and genotypes of PLGGs. Lastly, manual tumor segmentation is a time-consuming and costly task. In future, we will employ semi-automated or automated segmentation algorithms to achieve accurate and repeatable tumor segmentation.

## Conclusions

Our findings suggest that radiomic patterns are significantly associated with molecular biomarker (*BRAF* fusion) and able to predict molecular subgroups of PLGGs with a satisfying performance. Investigations with larger sample size are welcomed to further unravel the relationship between radiomics and molecular biomarkers/subgroups of PLGGs.

### Electronic supplementary material

Below is the link to the electronic supplementary material.

Supplementary Material 1

Supplementary Material 2

Supplementary Material 3

Supplementary Material 4

Supplementary Material 5

## Data Availability

All data generated or analyzed during this study are included in this published article.

## Abbreviations

PLGG: Pediatric low-grade glioma
CNS: Central nervous system
WHO: World Health Organization
PA: Pilocytic astrocytoma
A: Astrocytoma
O: Oligodendroglioma
OA: Oligoastrocytoma
PXA: Pleomorphic xanthoastrocytoma
AI: Artificial intelligence
ADC: Apparent diffusion coefficient
DWI: Diffusion-weighted imaging
FFPE: Formalin-fixed paraffin embedded
FISH: Fluorescence in situ hybridization
IHC: Immunohistochemistry
GLCM: Gray-level co-occurrence matrix
GLRLM: Gray-level run length matrix
GLSZM: Gray-level size zone matrix
GLDM: Gray-level dependence matrix
NGTDM: Neighborhood gray-tone difference matrix
LoG: Laplacian of Gaussian
ROC: Receiver operating characteristic
AUC: Area under the curve
ACC: Accuracy
SEN: Sensitivity
SPE: Specificity
DTI: Diffusion tensor imaging
PWI: Perfusion-weighted imaging

## Code Availability

The code scripts used in this article are deposited into github.com at https://github.com/PLGG2023/Randomforest-code and can be accessed and downloaded for free.
