# Supplementary material for: Radiomic features from multiparametric magnetic resonance imaging predict molecular subgroups of pediatric low-grade gliomas
Source: BMC Cancer. 2023 Sep 11;23:848. doi: 10.1186/s12885-023-11338-8 (PMC10496393; doi:10.1186/s12885-023-11338-8)
Supplement: Supplementary file 1 — Supplementary Material 1 [file 12885_2023_11338_MOESM1_ESM.docx]

**Supplementary Material**

**Image acquisition**

All patients in the local hospital were performed on either 1.5 T or 3.0 T clinical MR scanners. The 1.5 T scanners included Siemens Healthcare (Magnetom Avanto, Erlangen, Germany), and Philips Healthcare (Achieva, Best, Netherlands). The 3.0 T scanners included Siemens Healthcare (Magnetom Skyra/Verio/Trio TIM/Prisma, Erlangen, Germany), GE Healthcare (Discovery MR750/Signa HDxt, Milwaukee, WI, USA), and Philips Healthcare (Ingenia, Best, Netherlands). The brain imaging protocol includes the following sequences: (a) precontrast axial T1-weighted imaging (T1); (b) axial T2-weighted imaging (T2); (c) axial T2-weighted fluid-attenuated inversion recovery (FLAIR) imaging; (d) axial contrast-enhanced axial T1-weighted imaging (T1c); (e) diffusion-weighted imaging (DWI). The contrast-enhanced sequences were acquired immediately after intravenous administration of a 0.1 mmol/kg dose of gadolinium-based contrast agent (Gadopentetic Acid Dimeglumine Salt Injection, Magnevist, Bayer Healthcare, Berlin, Germany, or Gadoteric Acid Meglumine Salt Injection, Hengrui Healthcare, Jiangsu, China), followed by a 20-ml saline flush with an injection velocity of 2.0 ml/s. All DWI acquisitions were acquired before injection of the contrast agent and were used a spin echo single-shot echo-planar sequence including the values of b = 0 and b = 1000 s/mm^2^, with diffusion sensitizing gradients encoded in the x, y, and z directions. The corresponding ADC maps were generated with a monoexponential model on a voxel-by-voxel basis for all imaging planes using the Syngo workstation (Siemens Healthcare, Erlangen, Germany), or the Advantage workstation (Version 4.6, GE Healthcare, Milwaukee, WI, USA), or the workstation (Philips Healthcare, Best, Netherlands). Detailed information about the MR machines and imaging parameters are available in Supplementary Table 1.
