# Supplementary material for: Radiomic features from multiparametric magnetic resonance imaging predict molecular subgroups of pediatric low-grade gliomas
Source: BMC Cancer. 2023 Sep 11;23:848. doi: 10.1186/s12885-023-11338-8 (PMC10496393; doi:10.1186/s12885-023-11338-8)
Supplement: Supplementary file 2 — Supplementary Material 2 [file 12885_2023_11338_MOESM2_ESM.docx]

**Supplementary Table 1** MR imaging parameters in the local hospital

| MR system | GE Healthcare | | Siemens Healthcare | | | | | Philips Healthcare | |
| --- | --- | --- | --- | --- | --- | --- | --- | --- | --- |
|  | GE Discovery MR750 | GE Signa HDxt | Siemens  Verio | Siemens  Prisma | Siemens  Trio TIM | Siemens  Skyra | Siemens  Avanto | Philips Ingenia | Philips Achieva |
| Field strength (T) | 3 | 3 | 3 | 3 | 3 | 3 | 1.5 | 3 | 1.5 |
| T1w/T1c |  | |  | | | | |  | |
| TR (ms) | 1750-2358 | | 163-280 | | | | | 250-499 | |
| TE (ms) | 23-38 | | 2.46-4.76 | | | | | 2.302-15 | |
| TI (ms) | 705-780 | | N/A | | | | | N/A | |
| Section thickness (mm) | 5 | | 5 | | | | | 6 | |
| Image slice spacing (mm) | 1.5-2 | | 1.5-1.75 | | | | | 1 | |
| FA (°) | 90-111 | | 70-80 | | | | | 69-75 | |
| Number of averages/  Excitations | 1-2 | | 1 | | | | | 1 | |
| Pixel size (mm^2^) | 0.4688×0.4688 | | 0.4492×0.4492-0.8594×0.8594 | | | | | 0.4492×0.4492-0.575×0.575 | |
| Matrix | 320×224-288×288 | | 256×162-320×256 | | | | | 184×147-256×205 | |
| FOV (mm^2^) | 240×180-240×240 | | 220×172-240×240 | | | | | 230×183-230×185 | |
| ETL | 8-12 | | 1 | | | | | 1 | |
| T2w |  | |  | | | | |  | |
| TR (ms) | 3560-5848 | | 3600-6300 | | | | | 1953-5000 | |
| TE (ms) | 89-120 | | 85-125 | | | | | 70-100 | |
| TI (ms) | N/A | | N/A | | | | | N/A | |
| Section thickness (mm) | 5 | | 5 | | | | | 6 | |
| Image slice spacing (mm) | 1.5-2 | | 1.5-1.75 | | | | | 1 | |
| FA (°) | 90-142 | | 90-150 | | | | | 90 | |
| Number of averages/  Excitations | 1 | | 1-2 | | | | | 1-2 | |
| Pixel size (mm^2^) | 0.4688×0.4688 | | 0.3594×0.3594-0.75×0.75 | | | | | 0.3993×0.3993-0.5324×0.5324 | |
| Matrix | 384×256-512×512 | | 320×177-384×384 | | | | | 220×159-384×273 | |
| FOV (mm^2^) | 240×180-240×240 | | 220×162-240×240 | | | | | 230×183-230×189 | |
| ETL | 26-32 | | 16-28 | | | | | 16-17 | |
| FLAIR |  | |  | | | | |  | |
| TR (ms) | 8002-8500 | | 4500-8000 | | | | | 6000 | |
| TE (ms) | 156-173 | | 81-95 | | | | | 115-120 | |
| TI (ms) | 2000-2100 | | 1670-2372 | | | | | 2000-2250 | |
| Section thickness (mm) | 5 | | 5 | | | | | 6 | |
| Image slice spacing (mm) | 1.5-2 | | 1.5-1.75 | | | | | 1 | |
| FA (°) | 90-111 | | 130-150 | | | | | 90 | |
| Number of averages/  Excitations | 1 | | 1 | | | | | 1-2 | |
| Pixel size (mm^2^) | 0.4688×0.4688 | | 0.4297×0.4297-0.75×0.75 | | | | | 0.4492×0.4492-0.8984×0.8984 | |
| Matrix | 288×192-256×256 | | 256×150-320×224 | | | | | 176×117-328×168 | |
| FOV (mm^2^) | 240×240 | | 220×162-240×240 | | | | | 230×182-230×206 | |
| ETL | 1 | | 1-29 | | | | | 25-38 | |
| DWI |  | |  | | | | |  | |
| TR (ms) | 4800-6000 | | 3400-6200 | | | | | 2205-2798 | |
| TE (ms) | 74-88 | | 80-119 | | | | | 51-90 | |
| TI (ms) | N/A | | N/A | | | | | N/A | |
| Section thickness (mm) | 5 | | 5 | | | | | 6 | |
| Image slice spacing (mm) | 1.5-2 | | 1.5-1.75 | | | | | 1 | |
| FA (°) | 90 | | 90 | | | | | 90 | |
| Number of averages/  Excitations | 1 | | 1-3 | | | | | 1 | |
| Pixel size (mm^2^) | 0.9375×0.9375 | | 0.5990×0.5990-1.5132×1.5132 | | | | | 0.8984×0.8984-1.0268×1.0268 | |
| Matrix | 160×160 | | 152×152-220×220 | | | | | 112×89-152×114 | |
| FOV (mm^2^) | 240×240 | | 220×220-240×240 | | | | | 230×230 | |
| ETL | 1 | | 1-82 | | | | | 47-59 | |
| B value (s/mm^2^) | 0, 1000 | | 0, 1000 | | | | | 0, 1000 | |
