# Supplementary material for: Radiomic features from multiparametric magnetic resonance imaging predict molecular subgroups of pediatric low-grade gliomas
Source: BMC Cancer. 2023 Sep 11;23:848. doi: 10.1186/s12885-023-11338-8 (PMC10496393; doi:10.1186/s12885-023-11338-8)
Supplement: Supplementary file 5 — Supplementary Material 5 [file 12885_2023_11338_MOESM5_ESM.docx]

**Supplementary Table 4** The meanings of the 8 radiomic features constituting the radiomic model.

To fully characterize the image phenotypes within the tumor, we extracted radiomic features from not only the original medical images but also the transformed, or derived images by using wavelet or Laplacian of Gaussian (LoG) filters onto the original images. Finally, we extracted radiomic features from three types of images: original images, wavelet images, and LoG images. Wavelet images were obtained by applying wavelet transform on the original images. Wavelet transform can decouple informative textures by decomposing the original images into multiple low- and high-frequency components. Let *H* and *L* be a high-pass and low-pass wavelet function, respectively. Then, the eight decomposed images can be denoted as ***I****_HHH_*, ***I****_HHL_*, ***I****_HLH_*, ***I****_HLL_*, ***I****_LHH_*, ***I****_LHL_*, ***I****_LLH_*, ***I****_LLL_*, where the three subscripts meant the high- or low-pass filtering operations along *x*, *y* and *z* directions of the original 3D MR image. LoG images were obtained by applying LoG filtering operation on the original images. LoG performs two filtering operations, a Gaussian filtering, and a Laplacian filtering. Finally, four all-relevant features were selected for classification of molecular subgroups (low-risk group and intermediate/high-risk group). Meanwhile, four all-relevant features were selected for prediction of *BRAF* gene fusion. The feature selection results were summarized in **Table 2**. The meanings of these features are described as follows. These features defined below are in compliance with feature definitions as described by the Imaging Biomarker Standardization Initiative (IBSI), which are available in a separate document by Zwanenburg et al. (2016) [^[1]^](https://pyradiomics.readthedocs.io/en/latest/features.html#id3).

Supplementary Table 4-1: The meanings of four relevant features for classification of low-risk group and intermediate/high-risk group.

| Selected Features | Meanings |
| --- | --- |
| ADC_log.sigma.5.0.mm.3D_glcm_ClusterShade | Cluster Shade is a measure of the skewness and uniformity of the GLCM. A higher cluster shade implies greater asymmetry about the mean. |
| ADC_wavelet.LLH_gldm_DependenceEntropy | A measures method in dependence size in the image. |
| FLAIR_log.sigma.2.0.mm.3D_gldm_DependenceEntropy | A measures method in dependence size in the image. |
| T1_log.sigma.5.0.mm.3D_glrlm_GrayLevelVariance | GLV measures the variance in gray level intensity for the runs. |

Supplementary Table 4-2: The meanings of four relevant features for prediction of *BRAF* fusion.

| Selected Features | Meanings |
| --- | --- |
| ADC_wavelet.LLH_gldm_DependenceEntropy | A measures method in dependence size in the image. |
| T1c_original_firstorder_Minimum | A method for describing the distribution of voxel intensities within the image region. |
| T1c_wavelet.LLL_glszm_GrayLevelNonUniformity | Measures the similarity of gray-level intensity values in the image, where a lower GLN value correlates with a greater similarity in intensity values. |
| T1_log.sigma.5.0.mm.3D_glcm_ClusterProminence | Cluster Prominence is a measure of the skewness and asymmetry of the GLCM. A higher values implies more asymmetry about the mean while a lower value indicates a peak near the mean value and less variation about the mean. |

1. Zwanenburg A, Leger S, Vallières M, Löck S (2016) Image biomarker standardisation initiative - feature definitions. In eprint arXiv:1612.07003 [cs.CV].
